# Using Africa's protected area network to estimate the global population of a threatened and declining species: a case study of the Critically Endangered White‐headed Vulture *Trigonoceps occipitalis*

**DOI:** 10.1002/ece3.1931

**Published:** 2016-01-22

**Authors:** Campbell Murn, Peter Mundy, Munir Z. Virani, Wendy D. Borello, Graham J. Holloway, Jean‐Marc Thiollay

**Affiliations:** ^1^ Hawk Conservancy TrustAndoverHampshireSP11 8DYUK; ^2^ Centre for Wildlife Assessment and ConservationSchool of Biological SciencesUniversity of ReadingBerkshireRG6 6ASUK; ^3^ Department of Forest Resources and Wildlife ManagementNational University of Science and TechnologyBulawayoZimbabwe; ^4^ Ornithology SectionDepartment of ZoologyNational Museums of KenyaP.O Box 40658Nairobi00100Kenya; ^5^ The Peregrine Fund 5688 West Flying Hawk LaneBoiseIdaho83709; ^6^ BirdLife BotswanaPO Box 26691GaboroneBotswana; ^7^ Rue de la Rivière10220Rouilly ‐ SaceyFrance

**Keywords:** Decline, nest densities, protected areas, *Trigonoceps occipitalis*, White‐headed Vulture

## Abstract

The White‐headed Vulture *Trigonoceps occipitalis* (WhV) is uncommon and largely restricted to protected areas across its range in sub‐Saharan Africa. We used the World Database on Protected Areas to identify protected areas (PAs) likely to contain White‐headed Vultures. Vulture occurrence on road transects in Southern, East, and West Africa was adjusted to nests per km^2^ using data from areas with known numbers of nests and corresponding road transect data. Nest density was used to calculate the number of WhV nests within identified PAs and from there extrapolated to estimate the global population. Across a fragmented range, 400 PAs are estimated to contain 1893 WhV nests. Eastern Africa is estimated to contain 721 nests, Central Africa 548 nests, Southern Africa 468 nests, and West Africa 156 nests. Including immature and nonbreeding birds, and accounting for data deficient PAs, the estimated global population is 5475 ‐ 5493 birds. The identified distribution highlights are alarming: over 78% (*n* = 313) of identified PAs contain fewer than five nests. A further 17% (*n* = 68) of PAs contain 5 ‐ 20 nests and 4% (*n* = 14) of identified PAs are estimated to contain >20 nests. Just 1% (*n* = 5) of PAs are estimated to contain >40 nests; none is located in West Africa. Whilst ranging behavior of WhVs is currently unknown, 35% of PAs large enough to hold >20 nests are isolated by more than 100 km from other PAs. Spatially discrete and unpredictable mortality events such as poisoning pose major threats to small localized vulture populations and will accelerate ongoing local extinctions. Apart from reducing the threat of poisoning events, conservation actions promoting linkages between protected areas should be pursued. Identifying potential areas for assisted re‐establishment via translocation offers the potential to expand the range of this species and alleviate risk.

## Introduction

Vultures are threatened across many parts of the world (Ogada et al. 2012) and more than half (69%) have an unfavorable conservation status (BirdLife, 2015). Populations of three *Gyps* species in South Asia declined by more than 95% in the late 1990s due to incidental poisoning from the veterinary drug diclofenac (Prakash 1999; Green et al. 2004; Oaks et al. 2004) and populations of other vulture species in that region have also declined significantly (Cuthbert et al. 2006). Over large parts of Africa vultures are severely threatened and populations of most species are declining (Rondeau and Thiollay 2004; Thiollay 2007a; Ogada and Buij 2011; Virani et al. 2011; Krüger et al. 2014), and these ongoing declines mean that the conservation status of most species on the continent is now considered critical (Ogada et al. 2015).

The White‐headed Vulture *Trigonoceps occipitalis* (Burchell 1824) is a large and distinctive species that is widely distributed across sub‐Saharan Africa in a patchy distribution (Mundy et al. 1992). It is generally a solitary species and nests in isolated, possibly territorial pairs (Hustler and Howells 1988; Murn and Holloway 2014); only rarely are more than four or five birds reported to occur together (e.g. Culverwell 1985) and there are no published records of the species breeding outside protected areas. As a result the species is considered to be widespread but uncommon and also sensitive to increased human disturbance outside protected areas. In 2015 the category of risk assigned to the White‐headed Vulture by the IUCN increased from “Vulnerable” to “Critically Endangered” (BirdLife, 2015), which highlights the need to focus attention on the species and address its poor conservation status. In addition to addressing this unfavorable status, the White‐headed Vulture warrants attention due to it being distinctive in a number of ways. In addition to being monotypic (Lerner and Mindell 2005), the White‐headed Vulture exhibits a characteristic breeding biology (Murn and Holloway 2014), an unusual feeding ecology compared to other vulture species (Murn 2014) and is unique among African vultures in being sexually dimorphic (Mundy 1985). These unusual features emphasize the recognition of this species as a conservation priority (Lotz 2015).

The revised conservation status of the White‐headed Vulture began in 2007 and was due mainly to reports of vultures and other large birds of prey experiencing major declines during the previous two decades in West Africa (Thiollay 2001, 2006a,b, 2007a). For White‐headed Vultures, these declines exceeded 60% in protected areas, and the species was not recorded at all in rural areas (Thiollay 2006a,b, 2007b). More recently, a continental‐wide assessment indicated that the species has declined by as much as 97% in recent decades (Ogada et al. 2015) and this finding led directly to its conservation status being revised to “Critically Endangered” in 2015. However, that study assessed rates of decline rather than actual population estimates, and so assessments of actual White‐headed Vulture populations are few. From East Africa, recent work in Uganda (Pomeroy et al. 2015) indicates that between 44 and 187 White‐headed Vultures may exist in that country, and whilst this is currently the only population data available for the region, in Kenya major declines in abundance have been recorded (Virani et al. 2011). It has also been suggested that in common with most other vulture and eagle species, populations of White‐headed Vultures in Tanzania are experiencing long‐term declines (N. Baker, pers. comm.). Across much of southern Africa, where there were an estimated 430 pairs (Monadjem 2004), the White‐headed Vulture has been considered as restricted to protected areas for several decades (Steyn 1982; Hustler 1986; Mundy 1997; Simmons and Bridgeford 1997; Herremans and Herremans‐Tonnoeyr 2000). For example, the Kruger National Park (23°59′S, 31°36′E) and neighboring conservation areas have for some time held the largest population of the species in South Africa (Tarboton et al. 1987; Murn et al. 2013). However, in some countries, such as Botswana (Borello 1987) and Mozambique (Parker 1999, 2005), the species is considered to be widespread but uncommon and at low densities. Although data are scarce, it is likely that the species has suffered a range and population contraction in southern Africa (Tarboton and Allan 1984; Anderson 2000; Ferguson‐Lees and Christie 2001), though not to the same extent as in West Africa.

The existing global population estimate of 7000 – 12,000 White‐headed Vultures was made from a calculation that used averaged data from road transects and a proportional extrapolation to sub‐Saharan Africa, from southern Africa (Mundy et al. 1992). At the time, the authors indicated the difficulty of making a serious estimate of the species' numbers, and there are good reasons for this. Compared to other vultures in southern Africa the population status of the White‐headed Vulture is not well‐known, and in the rest of Africa its status is poorly known. Any data on the occurrence and status of White‐headed Vultures are limited, published infrequently and usually take the form of counts of birds made during road transects.

Despite a lack of population information, the association of White‐headed Vultures with protected areas observed in southern Africa has also been reported in West Africa (Thiollay 2006a,b, 2007b) and East Africa (Virani et al. 2011). This association is particularly the case for nests of breeding birds, even where individuals are seen outside protected areas (Pomeroy et al. 2015). As a result, there exists the potential to use the distribution and size of the African protected area network to estimate the global population of breeding White‐headed Vultures. This study uses a new method to re‐assess the global population of the White‐headed Vulture. We use data on the size, extent, and characteristics of Africa's protected area network combined with field data, published survey results and local information to revise the population estimate and, subsequently, examine the distribution and capacity of the African protected area network to maintain the global population of this species.

## Methods

### Global distribution of the White‐headed Vulture

The expected distribution of the White‐headed Vulture and the countries in which it is present was derived from a combination of sources. The detailed range map in Mundy et al. (1992) incorporated a range of historical and contemporary (at the time) field reports. The more recent IUCN Red List map (IUCN, 2014) updates this range map, but is essentially the same and makes no significant range expansions or contractions. The IUCN Red List species account provides a list of range countries in addition to the map. Web‐published accounts of birding trip reports and local sightings as well as information from historical published accounts were also utilized to assess the current distribution of the species.

### Selection and assessment of protected areas

Within the identified range countries, information on all protected areas was retrieved from the World Database on Protected Areas (IUCN & UNEP‐WCMC, 2012). A “protected area” (PA) is defined by the IUCN as: “A clearly defined geographical space, recognized, dedicated, and managed, through legal or other effective means, to achieve the long‐term conservation of nature with associated ecosystem services and cultural values” (Dudley 2008, p8). Six PA categories exist within this definition; each category describes broad differences in the interpretation of the definition. However, the IUCN categories were not suitable as a filter for selecting PAs relevant to White‐headed Vultures because not all recorded PAs have an associated IUCN category. Consequently, data on PA size, status, and location, in addition to the IUCN category were retrieved from the WDPA. This information was imported as shape files into a Geographical Information System for analysis.

All PAs have a specific designation (if not an IUCN category), but there is a wide range of them, and the WDPA list was filtered to determine the PAs that could realistically be expected to maintain nests of White‐headed Vultures. The specific designation of each PA enabled it to be grouped into one of three categories:

1. Protected areas with a nature and/or wildlife emphasis (e.g. National Parks, Nature Reserves, Wildlife Management Areas)
2. Areas emphasizing the protection of natural resources and their sustainable use (e.g. Forest Reserves, Classified Forests)
3. Unrelated areas (e.g. Marine Reserves, Fisheries)

Only confirmed PAs from the first category were selected; proposed PAs were not included. PAs from category two were not selected as PAs in this category are generally small and have a community/human emphasis. White‐headed Vultures are not noted as being associated with human activity and we considered it very unlikely for them to be nesting or even foraging on relatively small communal forest reserves in areas with established local communities. Table 1 lists the main PA designations and those that were selected for analysis. The reported sizes of the PAs in each country were standardized to square kilometres. Biosphere reserves and World Heritage Areas were excluded because these areas represent networks of existing PAs such as national parks and nature reserves. Similarly, areas listed under RAMSAR (the IUCN Wetlands Convention) were not included. Each of these network designations was checked to ensure that relevant PAs were not duplicated or deleted. Additional protected areas that were not listed, such as larger conservancies in southern Africa, were included where data were available.

**Table 1:** Protected area designations according to the World Database on Protected Areas (www.protectedplanet.net), showing protected areas that were included for analysis and categories that were excluded

Included	Excluded
National park	Community forest/village forest reserve
National reserve	State forest reserve or forest reserve
Strict nature reserve	Classified forest
Nature reserve	National forest priority area
Faunal reserve	Game reserve (<250 km^2^)
Wildlife reserve	Botanical reserve
Fauna and flora reserve	Special reserve
Partial fauna reserve	Partial reserve
Game reserve (>250 km^2^)	Marine park/marine reserve
Conservation area	Collaborative fishery management area
Wildlife sanctuary	Wetlands
Game sanctuary/game park	World heritage site/national heritage site
Wildlife management area	Biosphere reserve
Game management area	Protected landscape section
Controlled hunting area	Natural monument
Game controlled area	Sanctuary
Hunting reserve/safari area	Reforestation area
Game park/game farm	Unspecified protected area or “other”
	Recreational parks/resorts

PAs were also filtered according to size because the density and abundance of raptors reduces due to edge effects as the boundary of an area is approached (Herremans and Herremans‐Tonnoeyr 2000). Smaller PAs are affected proportionately more by edge effects and the size of a designated area can affect vulture presence; smaller areas can be associated with fewer vultures (Murn and Anderson 2008). White‐headed Vultures are generally considered to be territorial (Hustler and Howells 1988; Mundy et al. 1992), and for the purposes of data analysis in most areas a vulture territory was defined as 100 km^2^, which was based on existing estimates (Mundy 1982; Steyn 1982) and the mean nearest neighbor distance of the species (Murn and Holloway 2014). We also used data from the Serengeti (Pennycuick 1976) to define territory size as 400 km^2^ in East African savannas. For both territory size estimates, in addition to a 50 km^2^ buffer to account for edge effects, only PAs equivalent to double the expected territory size of White‐headed Vultures were selected. For these reasons, protected areas smaller than 250 km^2^ were excluded unless the area was part of a larger network of continuous protected areas. For the East African savanna estimate, any protected areas smaller than 850 km^2^ were excluded, again, unless the area was part of a larger network. Whilst White‐headed Vultures will sometimes occur outside protected areas, we assumed that this was only likely to happen if a nearby protected area contained breeding birds.

Larger PAs in Africa can contain over 40 pairs of White‐headed Vultures (Murn et al. 2013) and represent the most important locations for the species. We defined medium‐sized PAs as those containing more than 20 pairs of White‐headed Vultures and also considered these to be sites containing viable populations if their area integrity was maintained and connectivity to other PAs was feasible. To assess the degree of isolation of these medium‐sized areas we measured the shortest straight‐line distance between the PA boundary and the next nearest PA that met the size and selection criteria. The degree of isolation for smaller PAs was assessed in the same way.

PAs outside the White‐headed Vulture range were excluded. For example, in West Africa, PAs north of latitude 17°N and south of 7°30′N were excluded as out of range (Mundy et al. 1992; IUCN, 2014), as these areas are north of the Sahel (into the arid Saharan Zone) and south of the Sudanian Savanna (into moist Guinean Forests) respectively. PAs that were 200 km or more away from areas covered by existing range maps for the species, or for which more recent accounts (i.e. published accounts, bird atlas records, birding lists, or trip reports) could not be found, were also excluded.

### Estimation of White‐headed Vulture nest density

Each PA was assigned a nest density estimate (rating) based on a number of factors. Primarily this was historical and (where available) recent road transect data, but also adjusted for broad environmental variables such as rainfall (see Appendix 1). Baseline data for calculating densities were obtained for West Africa in Burkina Faso, Mali and Niger (Thiollay 2006a,b, 2007b), East Africa in Kenya and Tanzania (Virani et al. 2011, M. Z. Virani , J.‐M. Thiollay, D. L. Ogada and D. Pomeroy, unpubl. data) and southern Africa in Botswana (W.D. Borello, unpubl. data, C. Murn, unpubl. data). Density estimates were calculated from road transect data assuming a transect width of 2 km (i.e. birds sighted up to 1 km either side of line of travel). Given that in some areas a 2 km transect width will be too narrow (e.g. open plains), whilst in others it will be too wide (e.g. tree savanna or woodland), on balance we considered 2 km a reasonable distance to perform the calculations. For example, road transect data of 1.3 White‐headed Vultures/100 km corresponds to a density of 0.0065 birds/km^2^ by dividing the transect abundance (birds per 100 km) by the transect area (km^2^) thus: (1.3/200) = 0.0065.

The most recent population density estimates for White‐headed Vulture are from 2013 (Murn et al. 2013), and in order to standardize the densities and provide a population estimate across the entire range of White‐headed Vultures for 2013, we annualized the rate of change from studies with longitudinal data from more than one time period and projected to 2013. The rate of annual change was calculated between 1969–2004 for West Africa (Thiollay 2006a,b, 2007b) and between 1988–2005 for East Africa (Virani et al. 2011) and projected to 2013.

Not all White‐headed Vultures recorded during road transects will be breeding birds. We used a ratio to correct sighting densities (from road transects) into nest densities as follows. Nest density data with a high degree of accuracy from comprehensive ground and aerial surveys (Murn et al. 2013) were combined with ~30,000 km of road transect data from the same area (C. Murn, unpubl. data). Using age ratio data (number of adults vs. immature birds) obtained from these road transects, the number of birds seen was adjusted to the number of adults (54%) and this number adjusted to the proportion of adults that made a breeding attempt (75%) (Murn and Holloway 2014). We assumed that (a) the sighting density must be at least equal to, or exceed, nest density (birds are more easily seen than nests, there are more birds than nests, and birds are mobile) and (b) wherever White‐headed Vultures occurred at all, the ratio between the number of observed birds and number of nests would remain approximately the same, whether the species occurred at high or low densities or nonbreeding birds congregated. Therefore, using the example above, a road transect density of 0.0065 birds/km^2^ (1.3 birds/100 km) was corrected by a factor of 0.405 (0.54 × 0.75) to create a nest density of 0.00263 nests/km^2^. Whilst juvenile and immature birds may occur in any given PA, it was also assumed that a breeding pair would persist only in a PA large enough to accommodate a breeding pair at the regional nest density estimate. Therefore in any PA estimated to have less than one breeding pair, we assumed that birds were present but not breeding.

The total population was estimated by adding immature birds and nonbreeding adults to the number of breeding pairs. Mundy et al. (1992) suggested, for Cape Vultures *Gyps coprotheres* (Forster, 1798) that an additional 0.33 immature birds and nonbreeding adults exist per breeding adult. Based on the age ratio data of White‐headed Vultures observed during road transects (C. Murn, unpubl. data), we added 0.46 additional immature and nonbreeding adult birds to the number of breeding adults.

The nest density estimate was modified by region, country or specific PA according to published and unpublished information, local birding reports and information obtained from local ornithologists. Where local information was not available, we used one or other of the density estimates that were calculated for Kruger National Park (Murn et al. 2013) ‐ either the overall density for Kruger of 0.0037 nests/km^2^ or the estimate from the lower density area of 0.0018 nests/km^2^, according to the position within the range and whatever unpublished information was available. Descriptions of the density rating assigned to each region and the protected areas in each country can be found in the Supporting Information: Appendix 1. The entire list of selected PAs and the nest density assigned to them is located in the Supporting Information: Tables S1 – S4. For each protected area the estimated number of White‐headed Vulture pairs is the product of the density (described in Appendix 1) and the area of the PA.

### Projected White‐headed Vulture breeding populations of different sizes

To assess the protective capacity of the identified PA network, we calculated projected population scenarios for PAs containing hypothetical White‐headed Vulture populations starting with four, 10, 20, and 21 nests. For each scenario the census population was calculated, as above, by adding an additional 0.46 immature and nonbreeding adults per breeding adult – resulting in census populations of 12, 29, 58, and 61 birds. There are no survival and mortality data for White‐headed Vultures, so we utilized data from other vulture species (Piper et al. 1999; Monadjem et al. 2012) and used the following age‐specific annual survival parameters: Juvenile (1st year) 70%, Immature (2–4 years) 92%, Adult (5+ years) 98%. Corresponding population age‐class proportions based on road transect data (C. Murn, unpubl. data) were: Juvenile (27%), Immature (19%), Adult (54%). Annual productivity of each hypothetical population was calculated by adding 0.65 fledglings per nest (Hustler and Howells 1988; Murn and Holloway 2014) and annual mortality was subtracted using the age‐specific survival per age group. The effect of additive mortality was assessed by removing three, five, and seven additional birds from the census population annually. We then plotted population trajectory curves over a period of 30 years.

## Results

Across the countries in which the White‐headed Vulture is known to occur, the WDPA lists 4806 PAs covering a reported area of approximately 4,570,000 km^2^. Based on the selection criteria, 8.3% (*n* = 400) of these PAs covering 36.9% of the reported area (1,687,294 km^2^) were identified as potentially containing White‐headed Vulture nests. Many PAs (*n* = 4406) were excluded from the initial WDPA list and these were mostly relatively small Classified Forests, Forest Reserves or smaller community‐based natural resource reserves. Selected PAs were approximately 6.5 times larger than nonselected areas and 4.5 times larger than all PAs combined (Table 2). Size was listed for all National Parks and Category II PAs, but 13.8% (*n* = 663) of the listed PAs did not have a reported size, which meant they were missed by the initial selection criteria. Each of these areas was examined and 611 were in excluded categories (Table 1), seven were out of range, 21 were already represented by existing (larger) PAs, and 11 could not be explained. Each of the remaining 11 areas was assessed according to size and shape within the GIS shape files and a conservative estimate was made that these 11 PAs covered between 7000 and 10,000 km^2^. All the areas were isolated from other PAs, and so a low density estimator was used (0.0018 nests/km^2^) to conclude that these areas contained between 12 – 18 pairs of White‐headed Vultures.

**Table 2:** Number and size of areas in the range of the White‐headed Vulture in Africa and the number selected for population assessment

	Number of protected areas	Total area (km^2^)	Mean size (km^2^)
All protected areas	4806	4,570,034	951
Excluded protected areas	4406	2,882,740	654
Selected protected areas	400	1,687,294	4218

The number of selected PAs and their size varied significantly between countries. Each country in the range of the White‐headed Vulture was predicted to contain breeding birds, although in some cases the estimated number of pairs was very low despite the size of PA network. Based on the density estimates applied to each country (Table 3), the estimated breeding population across the range of the species was 1893 pairs. By excluding PAs in East Africa based on the larger WhV territory size of 400 km^2^, this estimate decreases by 30 pairs to 1863. Furthermore, by including the White‐headed Vultures potentially contained within the 7000 to 10,000 km^2^ of PAs without reported sizes (above) the estimated number of breeding pairs was 1875 to 1881. Adding nonbreeding adults and immature birds to the number of breeding pairs the estimated number of birds was 5475 ‐ 5493.

**Table 3:** Country‐specific totals and the estimated global population of White‐headed Vultures, as calculated by density in selected protected areas (not all protected areas in each country ‐ see text for details)

Country	Number of selected protected areas	Size of protected areas (km^2^)	Estimated number of White‐headed Vulture pairs	Mean nest density across all protected areas
Angola	5	64,080	57	0.0007
Benin	5	12,625	17	0.0010
Botswana	37	167,832	95	0.0008
Burkina Faso	10	29,003	20	0.0008
Burundi	2	908	1	0.0009
Cameroon	7	12,453	6	0.0005
Central African Republic	11	53,389	79	0.0012
Chad	7	109,830	3	0.0001
Congo, Dem Rep.	3	29,910	12	0.0004
Côte d'Ivoire	5	15,022	27	0.0016
Eritrea	3	5,006	2	0.0004
Ethiopia	33	182,650	88	0.0006
Gambia, The	2	110	1	0.0019
Ghana	4	10,499	15	0.0019
Guinea	3	7,376	4	0.0014
Guinea‐Bissau	4	3,771	4	0.0011
Kenya	24	47,847	34	0.0006
Malawi	7	10,363	2	0.0002
Mali	9	25,630	15	0.0009
Mozambique	18	91,300	158	0.0019
Namibia	7	33,970	40	0.0020
Niger	5	103,781	2	0.0003
Nigeria	17	22,134	14	0.0006
Rwanda	2	1,930	2	0.0009
Senegal	5	22,555	24	0.0010
Somalia	2	5,540	5	0.0009
South Africa	12	37,813	82	0.0021
Sudan and South Sudan	14	112,350	88	0.0007
Tanzania	46	174,959	489	0.0022
Togo	3	3,856	7	0.0014
Uganda	13	16,308	12	0.0007
Zambia	46	220,156	400	0.0016
Zimbabwe	29	47,969	94	0.0020
Total (34 countries)	400	1,687,294	1893	0.0011

**Figure 1:**
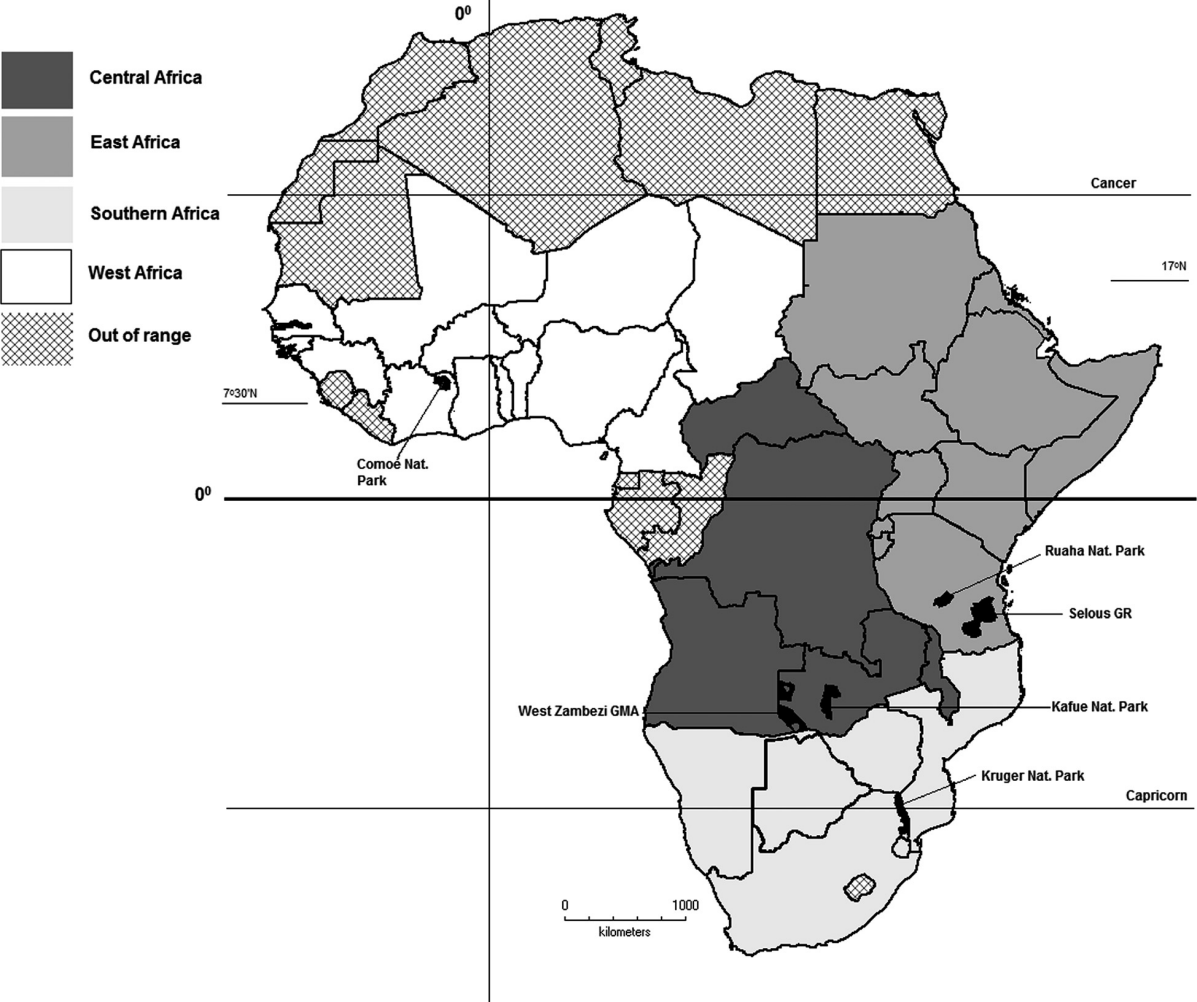
Countries assessed for the global White‐headed Vulture population. Five protected areas with more than 40 nests in East, Central and Southern Africa are indicated. Comoé National Park in West Africa is estimated to contain the largest population (~20 nests) in that region.

Based on the identified PA distribution, the range of the White‐headed Vulture is highly fragmented. Of the 400 PAs identified during the assessment, 78.3% (*n* = 313) were predicted to contain fewer than five nests. Substantial populations (more than 40 nests) were predicted to occur in five locations: Selous Game Reserve (08°30′S 37°36′E) and Ruaha National Park (07°24′S 34°42′E) in Tanzania, Kafue National Park (14°53′S 25°45′E) and West Zambezi Game Management Area (16°12′S 22°28′E) in Zambia, and Kruger National Park in South Africa (Fig. 1). No PAs in West Africa were predicted to have more than 40 nests and only Comoé National Park (09°12′N 03°39′W) in Côte d'Ivoire (Fig. 1) was predicted to have more than 20 nests. Of the larger PAs (20–40 nests), 32% (*n* = 6) were isolated by more than 100 km from the next nearest PA of a size within the selection criteria. Table 4 lists the number and percentage of PAs predicted to contain various numbers of White‐headed Vulture nests and Table S1 (Supporting Information) lists the calculated nesting density and predicted number of breeding pairs for each PA that was selected and assessed.

**Table 4:** Projected White‐headed Vulture breeding populations and the number of protected areas in which they occur in different regions of Africa. Protected area selection was based on size, designation and position within the range of the species. Figures in parentheses for East Africa incorporate territory size data from Pennycuick (1976). See text for details

	Region	West	East	Central	Southern	Total
Number of protected areas		86	139 (103)	72	103	400 (364)
Pairs		156	721 (691)	548	468	1893 (1863)
Number of protected areas with						
<5 pairs		79	116 (80)	42	76	313 (277)
5–20 pairs		6	17	22	23	68
20–40 pairs		1	4	6	3	14
>40 pairs		0	2	2	1	5

**Figure 2:**
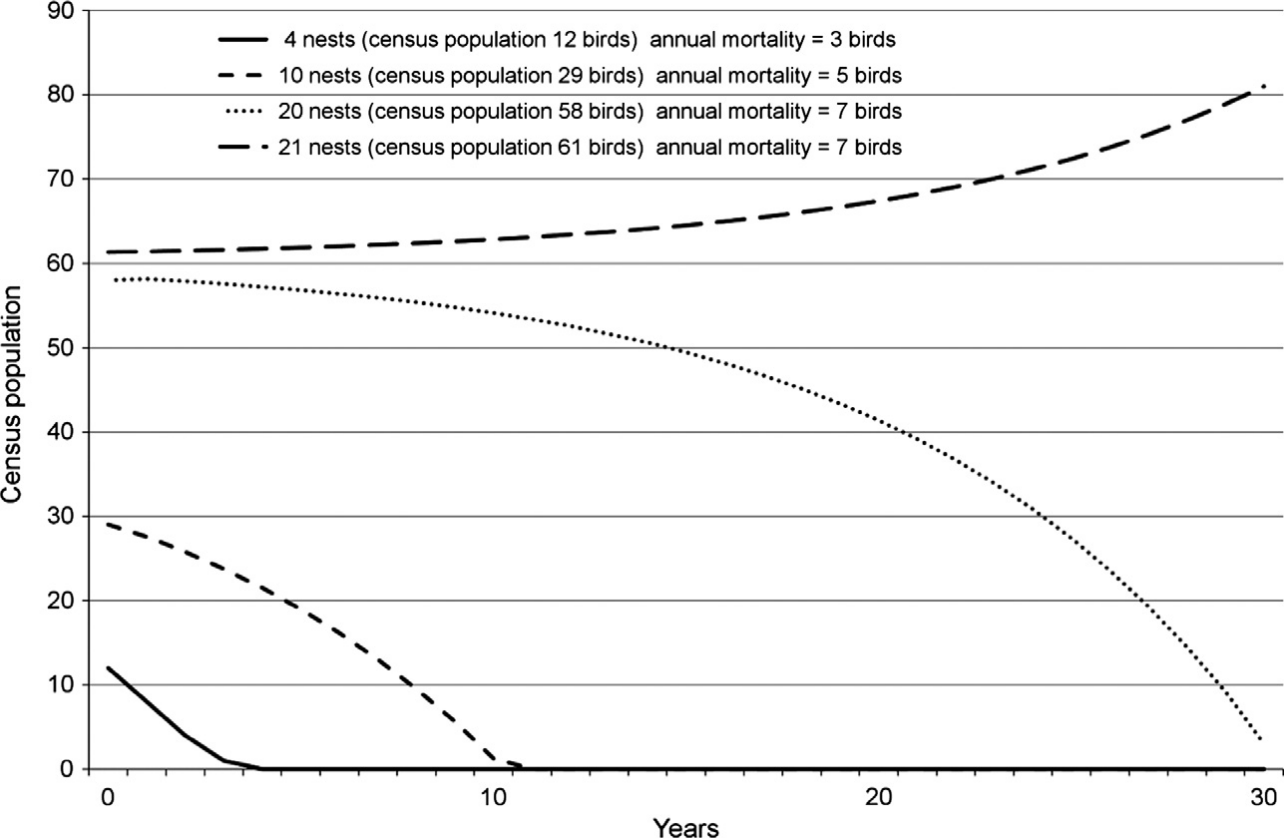
Rates of change for hypothetical White‐headed Vulture populations of four sizes (number of nests) based on varying amounts of additive annual mortality.

Based on the survival, age‐proportion, and productivity parameters outlined above, Figure 2 shows the trajectories calculated for four hypothetical White‐headed Vulture populations. In the absence of any additive mortality, White‐headed Vulture populations in all scenarios were calculated either to remain stable or to increase. However, small populations (five to 10 nests, 12–30 birds) declined rapidly to zero with a small amount of extra mortality (three‐five adult deaths per annum). Larger populations were more robust to some additive mortality and based on the parameters used, PAs with more than 20 nests showed an increasing population size if additive mortality remained fewer than eight birds per annum (Fig. 2).

## Discussion

Like the previous estimate (Mundy et al. 1992), the global population figure presented here is an estimate based on extrapolation. However, we feel that a revised figure is justified on the basis of: (1) the alarming and significant declines of vultures across most of Africa (Thiollay 2007a; Virani et al. 2011; Ogada et al. 2015); (2) the inclusion of new methods (PA selection process, region‐ and country‐specific nest density estimates, utilization of regional and local information (published accounts, birding trip reports, etc.)); (3) the fact that the still widely‐used and cited previous estimate is old and very likely to be inaccurate and higher than the current situation for the species.

During the process it was necessary to rely on the assumption that each of the selected PA contains White‐headed Vultures and that nonprotected areas do not. Across most of West Africa the second part of this assumption is likely to be reasonable, given the declines of many raptor species there, and the reported density for White‐headed Vultures outside PAs being zero (Rondeau and Thiollay 2004; Thiollay 2006a,b, 2007b). The assumption can be met with moderate conviction in southern Africa (Steyn 1982; Hustler 1986; Mundy 1997), whilst studies in Kenya (Virani et al. 2011) and records from the Tanzanian Bird Atlas (N. Baker, pers. comm.) suggest the pattern of White‐headed Vultures and PAs is occurring in East Africa. It therefore seems likely that a similar pattern of occurrence is repeating across other parts of the White‐headed Vulture's range.

There are exceptions. In Uganda the occurrence of White‐headed Vultures in pastoral areas, where habitat remains largely intact but domestic cattle have replaced wildlife, is only slightly less than in adjacent protected areas (Pomeroy et al. 2015). A report from Angola (Mendelsohn 2013) notes that in and around Kameia National Park in eastern Angola, White‐headed Vultures are seen regularly, appear to be reasonably numerous and are encountered at the same rate inside and outside the national park. In remote areas the distinction between protected and nonprotected areas is not always clear, and large parts of Africa have not undergone significant habitat change or development. In these areas, the rate at which species like White‐headed Vultures are encountered may not differ between protected and nonprotected areas, as has been found for other species elsewhere (Barnes et al. 2015). However, these situations are rare and a contemporary report also from Angola (Thiollay 2013) highlights that during a recent birding trip, there were no vultures seen at all (apart from Palm‐nut Vulture *Gypohierax angolensis* (Gmelin, 1788)) in much of western Angola, which has comparatively higher human populations and associated habitat change. It is variations such as these that warrant selecting only for dedicated (as much as possible) wildlife areas in the PA network (Table 1), on the basis that the bird is not noted as being associated with human activity and is very unlikely to be nesting or even foraging on relatively small communal forest reserves in areas with established local communities.

A potential criticism of the method followed here is that too many PAs have been excluded from the analysis. Whilst it is possible that some PAs containing White‐headed Vultures were excluded during the selection process, it is also likely that other areas without the species, or with very low densities, have been included. An additional source of error is the variation in PA network between countries. For example, the estimate of 400 pairs in Zambia is higher than most other countries and is due to Zambia having a very large protected area network (>220,000 km^2^). However, any error in the global estimate is likely to be toward an inflated figure, as not all of Zambia's Wildlife Management Areas will contain White‐headed Vultures at the estimated density (Roxburgh and McDougall 2012; R. McDougall, pers. comm.). Similarly, Senegal has very few reports of White‐headed Vultures and recorded densities are very low (Petersen et al. 2007), yet the process followed here estimates that country as having 24 pairs, or approximately 70 birds. Overall, we would contend that the global estimate produced here represents a best‐case scenario.

The mean density for each country (Table 3) is thus more a reflection of the PA network and composition, rather than a direct measure of White‐headed Vulture density in each country – variations in density should not be used to assess the “suitability” of any given country for White‐headed Vultures. Countries with larger estimated totals are likely to have reasonable populations of White‐headed Vultures – if for no other reason than the protected area network in these countries is extensive. The population estimate for Botswana (95 pairs) is likely to be high, given the generally low density of the bird in this country, but there are more than 150,000 km^2^ of PAs potentially containing White‐headed Vultures in the country. Similarly, Tanzania and Zambia hold significant populations based on their very large PA networks. Another criticism of the process is that it over‐simplifies the variation in occurrence that a species range approaching five million square kilometres would contain. We contend that without a comprehensive aerial survey over millions of square kilometres, some method of estimation via extrapolation must be used. Indeed, such methods are used for other conservation‐dependent species that occur at low densities (Henschel et al. 2014), are cryptic or elusive (Hebblewhite et al. 2011) or occur over large areas (Greve et al. 2011). A “one size fits all” approach to estimating density is clearly inappropriate for a species with such a large range, but by taking regional, national, and in some cases local approaches to estimating densities, we consider the process followed here to be sufficiently detailed. Furthermore, with updated road transect data (e.g. Pomeroy et al. 2015) and/or actual nest densities from specific areas, the population estimates calculated here are directly comparable with future data, whilst detailed investigations in each country would provide even more salient comparisons.

### What future for the White‐headed Vulture?

Negative changes to the conservation status of several African vulture species (African White‐backed Vulture *Gyps africanus* (Salvadori, 1865), Rüppell's Vulture *Gyps rueppellii* (Brehm, 1852) and Hooded Vulture *Necrosyrtes monachus* (Temminck, 1823) were all listed as “Critically Endangered” by the IUCN in 2015) and the recent description of rapidly declining vulture populations across the continent overall (Ogada et al. 2015) would support an argument that the estimates provided here could be too high for the countries with relatively large populations, and particularly for smaller countries. Swaziland, for example, no longer has any breeding White‐headed Vultures in its parks or reserves (A. Monadjem, pers. comm.).

Overall, based on the global population estimate presented here and assuming the validity of the previous estimate (Mundy et al. 1992), the population of White‐headed Vultures has reduced by 27–60% over the last 25 years. Alone, this would justify a re‐assessment of an existing conservation status of this species to “Endangered”, based on the IUCN's Red List criteria (IUCN, 2014). However, the large (>95%) and long‐term declines reported for this species (Ogada et al. 2015) across most of Africa over recent decades, combined with a range of ongoing threats that include poisoning (Kendall and Virani 2012; Roxburgh and McDougall 2012), harvesting for the animal trade (Groom et al. 2013; McKean et al. 2013) and electrocution (Anderson and Kruger 1995; Angelov et al. 2013) further supports the recent revision of the conservation status of this species to “Critically Endangered” (BirdLife, 2015).

Irrespective of any changes to the Red List status, the prognosis for the current White‐headed Vulture population appears poor. Globally, the small breeding population of White‐headed Vultures is fragmented and vulnerable to stochastic events, particularly events related to additive mortality from the causes noted above. There is a very high level of extinction risk for breeding populations in the majority of PAs (>75%), which contain five or fewer nests of White‐headed Vultures; with only moderate levels of increased mortality these populations are likely to disappear (Fig. 2). A further 17% (*n* = 68) of PAs have estimated populations below 20 nests and the persistence of these populations is also highly vulnerable to moderately increased mortality. Together, these two PA categories hold 57% of the breeding White‐headed Vulture population. More optimistically, the network of larger PAs in which the bird occurs potentially offers some buffering for the population, which is one of the main roles of the protected area network (Gaston et al. 2008), and most of the large and important (for White‐headed Vultures) PAs are also recognized as important for many other taxa (Wegmann et al. 2014). However, despite this, many PAs in Africa are suffering the effects of human disturbance. For example, the integrity of many PAs across Africa is threatened by large‐scale development projects; more than 400 PAs are scheduled to be affected by planned road developments alone (Laurance et al. 2015), and particularly in West Africa and parts of East Africa, large mammal populations in PAs have declined over recent decades (Craigie et al. 2010), primarily due to human impacts. That vulture populations in general have declined significantly over the same period (Ogada et al. 2015) suggests that reduced PA integrity has a negative impact on vultures, and for the disturbance‐sensitive White‐headed Vulture, this impact is likely to be greater.

Optimism about the existence of substantial White‐headed Vulture populations in larger PAs must also be tempered by the fact that there are very few of them (five) and that very little is known about White‐headed Vulture movement ecology; the small number of ring recoveries (Oatley et al. 1998) that do exist indicate limited dispersal distances of less than 150 km over three years. Despite a generally unfavorable environment for White‐headed Vultures outside many PAs, preliminary tracking studies (A. Botha & C. Murn, unpubl. data; B. Garbett et al. *in litt*.) and reports from the field (Mendelsohn 2013) indicate that White‐headed Vultures are not completely restricted to PAs and do occur outside them during the course of their foraging (P. Mundy, pers. obs.). These movements may occur regularly, and whilst the distances travelled may be less than other vultures (Phipps et al. 2013), inherent risks remain. Protected areas themselves are not without risks to vultures (Groom et al. 2013), and in the areas outside them these are likely to be higher.

The threats facing vultures in Africa are now well‐recognized (Ogada et al. 2015) and concerted efforts from international‐level agreement downwards are required to address these issues. There is much still to be discovered about the White‐headed Vulture, in particular accurate estimates of survival and mortality and a detailed understanding of its movement ecology in the light of causes of mortality. Its reliance on and association with protected areas compared to other vultures has yet to be explained. Apart from efforts aimed at changing the environmental and cultural practices that cause the main threats to vultures, we emphasize the need to maintain protected area integrity and also identify new potentially viable protected areas for this and other vulture species. It is within this existing and potential PA network that opportunities for the conservation of this species will proceed. Away from these areas, and given the population decline of White‐headed Vultures, the potential and logistics of ex situ conservation efforts such as captive breeding programmes could be investigated.

## Conflict of Interest

None declared.

## Supporting information


**Table S1**. Number of White‐headed Vulture nests in selected protected areas in Southern Africa.
**Table S2**. Number of White‐headed Vulture nests in selected protected areas in East Africa.
**Table S3**. Number of White‐headed Vulture nests in selected protected areas in Central Africa.
**Table S4**. Number of White‐headed Vulture nests in selected protected areas in West Africa.

**Appendix S1.** Regional and country specific ratings for White‐headed Vulture nest densities.

## References

[ece31931-bib-0001] Anderson, M. D. 2000 Whiteheaded Vulture Pp. 79–81 *in* Barnes K. N., ed. The Eskom red data book of birds of South Africa, Lesotho and Swaziland. BirdLife South Africa, Johannesburg.

[ece31931-bib-0002] Anderson, M. D. , and R. Kruger . 1995 Power line electrocution of eighteen African White‐backed Vultures. Vulture News 32:16–18.

[ece31931-bib-0003] Angelov, I. , I. Hashim , and S. Oppel . 2013 Persistent electrocution mortality of Egyptian Vultures *Neophron percnopterus* over 28 years in East Africa. Bird Conserv. Int. 23:1–6.

[ece31931-bib-0004] Barnes, M. , J. K. Szabo , W. K. Morris , and H. Possingham . 2015 Evaluating protected area effectiveness using bird lists in the Australian Wet Tropics. Divers. Distrib. 21:368–378.

[ece31931-bib-0005] BirdLife . 2015 BirdLife International (2015) IUCN Red List for Birds. Available at: www.birdlife.org (accessed Nov 2015)

[ece31931-bib-0006] Borello, W. D. 1987 Vulture distribution in Botswana. Babbler 13:11–25.

[ece31931-bib-0012] Burchell, W. J. 1824 Travels in the interior of southern Africa. Longman, London.

[ece31931-bib-0014] Craigie, I. D. , J. E. M. Baillie , A. Balmford , C. Carbone , B. Collen , R. E. Green , et al. 2010 Large mammal population declines in Africa's protected areas. Biol. Conserv. 143:2221–2228.

[ece31931-bib-0015] Culverwell, J. 1985 A shoal of fish eagles. Afr. Wildl. 39:248.

[ece31931-bib-0016] Cuthbert, R. , R. E. Green , S. Ranade , S. Saravanan , D. J. Pain , V. Prakash , et al. 2006 Rapid population declines of Egyptian vulture *Neophron percnopterus* and red‐headed vulture *Sarcogyps calvus* in India. Anim. Conserv. 9:349–354.

[ece31931-bib-0017] Dudley, N. 2008 Guidelines for applying protected area management categories. IUCN, Gland, Switzerland.

[ece31931-bib-0018] Ferguson‐Lees, J. , and D. A. Christie . 2001 Raptors of the world. Christopher Helm, London.

[ece31931-bib-0019] Gaston, K. J. , S. F. Jackson , L. Cantú‐Salazar , and G. Cruz‐Piñón . 2008 The ecological performace of protected areas. Annu. Rev. Ecol. Evol. Syst. 39:93–113.

[ece31931-bib-0024] Green, R. E. , I. Newton , S. Shultz , A. A. Cunningham , M. Gilbert , D. J. Pain , et al. 2004 Diclofenac poisoning as a cause of vulture population declines across the Indian subcontinent. J. Appl. Ecol. 41:793–800.

[ece31931-bib-0025] Greve, M. , S. L. Chown , B. J. van Rensburg , M. Dallimer , and K. J. Gaston . 2011 The ecological effectiveness of protected areas: a case study for South African birds. Anim. Conserv. 14:295–305.

[ece31931-bib-0026] Groom, R. J. , E. Gandiwa , P. Gandiwa , and H. J. van der Westhuizen . 2013 A mass poisoning of White‐backed and Lappet‐faced vultures in Gonarezhou National Park. Honeyguide 59:5–9.

[ece31931-bib-0028] Hebblewhite, M. , D. G. Miquelle , A. A. Murzin , V. V. Aramilev , and D. G. Pikunov . 2011 Predicting potential habitat and population size for reintroduction of the Far Eastern leopards in the Russian Far East. Biol. Conserv. 144:2403–2413.

[ece31931-bib-0029] Henschel, P. , L. Coad , C. Burton , B. Chataigner , A. Dunn , D. MacDonald , et al. 2014 The lion in West Africa is critically endangered. PLoS ONE 9:e83500. 24421889 10.1371/journal.pone.0083500 PMC3885426

[ece31931-bib-0030] Herremans, M. , and D. Herremans‐Tonnoeyr . 2000 Land use and the conservation status of raptors in Botswana. Biol. Conserv. 94:31–41.

[ece31931-bib-0031] Hustler, K. 1986 A revised checklist of the birds of Hwange National Park. Honeyguide 32:68–87.

[ece31931-bib-0032] Hustler, K. , and W. W. Howells . 1988 Breeding biology of the Whiteheaded Vulture in Hwange National Park, Zimbabwe. Ostrich 59:21–24.

[ece31931-bib-0033] IUCN . 2014 IUCN Red List of Threatened Species. Version 2014.2. Available at: www.iucnredlist.org (accessed January 2015)

[ece31931-bib-0034] IUCN & UNEP‐WCMC . 2012 The World Database on Protected Areas (WDPA). Sept 2012. Cambridge (UK): UNEP World Conservation Monitoring Centre. Available at: www.protectedplanet.net (accessed September 2012

[ece31931-bib-0035] Kendall, C. J. , and M. Z. Virani . 2012 Assessing mortality of African vultures using wing tags and GSM‐GPS transmitters. J. Raptor Res. 46:135–140.

[ece31931-bib-0036] Krüger, S. C. , D. G. Allan , A. R. Jenkins , and A. Amar . 2014 Trends in territory occupancy, distribution and density of the Bearded Vulture *Gypaetus barbatus* meridionalis in southern Africa. Bird Conserv. Int. 24:162–177.

[ece31931-bib-0037] Laurance, W. F. , S. Sloan , L. Weng , and J. A. Sayer . 2015 Estimating the environmental costs of Africa's massive ‘development corridors’. Curr. Biol. 25:1–7. 26628009 10.1016/j.cub.2015.10.046

[ece31931-bib-0038] Lerner, H. R. L. , and D. P. Mindell . 2005 Phylogeny of eagles, Old World vultures, and other Accipitridae based on nuclear and mitochondrial DNA. Mol. Phylogenet. Evol. 37:327–346. 15925523 10.1016/j.ympev.2005.04.010

[ece31931-bib-0039] Lotz, C. 2015 The most urgent bird conservation priorities in Africa and its islands. Bull. Afr. Bird Club 22:92–96.

[ece31931-bib-0041] McKean, S. , M. Mander , N. Diedrichs , L. Ntuli , K. Mavundla , V. Williams , et al. 2013 The impact of traditional use on vultures in South Africa. Vulture News 65:15–36.

[ece31931-bib-0042] Mendelsohn, J. 2013 Vultures in Angola. Vulture News 63:71–72.

[ece31931-bib-0045] Monadjem, A. 2004 White‐headed Vulture *Trigonoceps occipitalis* Pp. 34–39 *in* Monadjem A., Anderson M. D., Piper S. E., Boshoff A. F.,ed. The Vultures of Southern Africa ‐ Quo vadis? Proceedings of a workshop on vulture research and conservation in southern Africa. Birds of Prey Working Group, Johannesburg.

[ece31931-bib-0046] Monadjem, A. , A. Botha , and C. Murn . 2012 Survival of the African white‐backed vulture *Gyps africanus* in north‐eastern South Africa. Afr. J. Ecol. 51:87–93.

[ece31931-bib-0047] Mundy, P. J. 1982 The comparative biology of southern African vultures. Vulture Study Group, Johannesburg.

[ece31931-bib-0048] Mundy, P. J. 1985 Sexual dimorphism of the African White‐Headed Vulture *Trigonoceps occipitalis* . Ibis 127:116–119.

[ece31931-bib-0049] Mundy, P. J. 1997 White‐headed Vulture *Trigonoceps occipitalis* Pp. 164–165 *in* Harrison J. A., Allan D. G., Underhill L. G., Herremans M., Tree A. J., Parker V., Brown C. J., ed. The atlas of southern African birds. Volume 1: non‐passerines. BirdLife South Africa, Johannesburg.

[ece31931-bib-0050] Mundy, P. , D. Butchart , J. Ledger , and S. Piper . 1992 The Vultures of Africa. Academic Press, London.

[ece31931-bib-0051] Murn, C. 2014 Observations of predatory behavior by White‐headed Vultures. J. Raptor Res. 48:2.

[ece31931-bib-0052] Murn, C. , and M. D. Anderson . 2008 Activity patterns of African White‐backed Vultures *Gyps africanus* in relation to different land‐use practices and food availability. Ostrich 79:191–198.

[ece31931-bib-0053] Murn, C. , and G. J. Holloway . 2014 Breeding biology of the White‐headed Vulture *Trigonoceps occipitalis* in Kruger National Park, South Africa. Ostrich 85:125–130.

[ece31931-bib-0054] Murn, C. , L. Combrink , G. S. Ronaldson , C. Thompson , and A. Botha . 2013 Population estimates of three vulture species in Kruger National Park, South Africa. Ostrich 84:1–9.

[ece31931-bib-0056] Oaks, J. L. , M. Gilbert , M. Z. Virani , R. T. Watson , C. U. Meteyer , B. A. Rideout , et al. 2004 Diclofenac residues as the cause of vulture population decline in Pakistan. Nature 427:630–633. 14745453 10.1038/nature02317

[ece31931-bib-0057] Oatley, T. B. , H. D. Oschadleus , R. A. Navarro , and L. G. Underhil l. 1998 Review of ring recoveries of birds of prey in southern Africa: 1948‐1998. Endangered Wildlife Trust, Johannesburg.

[ece31931-bib-0058] Ogada, D. L. , and R. Buij . 2011 Large declines of the Hooded Vulture *Necrosyrtes monachus* across its African range. Ostrich 82:101–113.

[ece31931-bib-0059] Ogada, D. L. , F. Keesing , and M. Z. Virani . 2012 Dropping dead: causes and consequences of vulture population declines worldwide. Ann. N. Y. Acad. Sci. 1249:57–71. 22175274 10.1111/j.1749-6632.2011.06293.x

[ece31931-bib-0060] Ogada, D. L. , P. Shaw , R. L. Beyers , R. Buij , C. Murn , J. M. Thiollay , et al. 2015 Another continental vulture crisis: Africa's vultures collapsing toward extinction. Conserv. Lett. . Another Continental Vulture Crisis: Africa's Vultures Collapsing toward Extinction. Conservation Letters. doi: doi.org/10.1111/conl.12182.

[ece31931-bib-0062] Parker, V. 1999 The atlas of the birds of Sul do Save, southern Mozambique. Avian Demography Unit and Endangered Wildlife Trust, Cape Town and Johannesburg.

[ece31931-bib-0063] Parker, V. 2005 The atlas of the birds of central Mozambique. Endangered Wildlife Trust and Avian Demography Unit, Johannesburg and Cape Town.

[ece31931-bib-0064] Pennycuick, C. J. 1976 Breeding of the lappet‐faced and white‐headed vultures (*Torgos tracheliotus* Forster and *Trigonoceps occipitalis* Burchell) on the Serengeti Plains, Tanzania. East Afr. Wildl. J. 14:67–84.

[ece31931-bib-0065] Petersen, B. S. , K. D. Christensen , and F. P. Jensen . 2007 Bird population densities along two precipitation gradients in Senegal and Niger. Malimbus 29:101–121.

[ece31931-bib-0066] Phipps, W. L. , S. G. Willis , K. Wolter , and V. Naidoo . 2013 Foraging ranges of immature African White‐backed Vultures (*Gyps africanus*) and their use of protected areas in southern Africa. PLoS ONE 81:e52813. 23382824 10.1371/journal.pone.0052813 PMC3559650

[ece31931-bib-0067] Piper, S. E. , A. F. Boshoff , and H. A. Scott . 1999 Modelling survival rates in the Cape Griffon Gyps coprotheres, with emphasis on the effects of supplementary feeding. Bird Study 46:230–238.

[ece31931-bib-0068] Pomeroy, D. , P. Shaw , M. Opige , G. Kaphu , D. L. Ogada , and M. Z. Virani . 2015 Vulture populations in Uganda: using road survey data to measure both densities and encounter rates within protected and unprotected areas. Bird Conserv. Int. 25:399–414.

[ece31931-bib-0069] Prakash, V. 1999 Status of vultures in Keoladeo National Park, Bharatpur, Rajasthan, with special reference to population crash in *Gyps* species. J. Bombay Nat. Hist. Soc. 96:365–378.

[ece31931-bib-0070] Rondeau, G. , and J. M. Thiollay . 2004 West African vulture decline. Vulture News 51:13–31.

[ece31931-bib-0071] Roxburgh, L. , and R. McDougall . 2012 Vulture poisoning incidents and the status of vultures in Zambia and Malawi. Vulture News 62:33–39.

[ece31931-bib-0073] Simmons, R. E. , and P. Bridgeford . 1997 The status and conservation of vultures in Namibia Pp. 67–75 *in* Boshoff A. F., Anderson M. D., Borello W. D., ed. Vultures in the 21st Century. Vulture Study Group, Johannesburg.

[ece31931-bib-0074] Steyn, P. 1982 Birds of Prey of southern Africa, their identification and life histories. David Philip, Cape Town.

[ece31931-bib-0075] Tarboton, W. R. , and D. G. Allan . 1984 The status and conservation of birds of prey in the Transvaal. Transvaal Museum Monograph 3:1–115.

[ece31931-bib-0076] Tarboton, W. R. , M. I. Kemp , and A. C. Kemp . 1987 Birds of the transvaal. Transvaal Museum, Pretoria.

[ece31931-bib-0078] Thiollay, J. M. 2001 Long‐term changes of raptor populations in northern Cameroon. J. Raptor Res. 35:173–186.

[ece31931-bib-0079] Thiollay, J. M. 2006a Large bird declines with increasing human pressure in savanna woodlands (Burkina Faso). Biodivers. Conserv. 15:2085–2108.

[ece31931-bib-0080] Thiollay, J. M. 2006b The decline of raptors in West Africa: long‐term assessment and the role of protected areas. Ibis 148:240–254.

[ece31931-bib-0081] Thiollay, J. M. 2007a Raptor population decline in West Africa. Ostrich 78:405–413.

[ece31931-bib-0082] Thiollay, J. M. 2007b Raptor declines in West Africa: comparisons between protected, buffer and cultivated areas. Oryx 41:322–329.

[ece31931-bib-0083] Thiollay, J. M. 2013 Has Angola lost all its vultures? Vulture News 63:68–70.

[ece31931-bib-0084] Virani, M. Z. , C. Kendall , P. Njoroge , and S. Thomsett . 2011 Major declines in the abundance of vultures and other scavenging raptors in and around the Masai Mara ecosystem, Kenya. Biol. Conserv. 144:746–752.

[ece31931-bib-0086] Wegmann, M. , L. Santini , B. Leutner , K. Safi , D. Rocchini , M. Bevanda , et al. 2014 Role of African protected areas in maintaining connectivitiy for large mammals. Philos. Trans. R. Soc. Lond. B Biol. Sci. 369:20130193. 24733948 10.1098/rstb.2013.0193 PMC3983928

