# Supplementary material for: Using Africa's protected area network to estimate the global population of a threatened and declining species: a case study of the Critically Endangered White‐headed Vulture Trigonoceps occipitalis
Source: Ecol Evol. 2016 Jan 22;6(4):1092–103. doi: 10.1002/ece3.1931 (PMC4761783; doi:10.1002/ece3.1931)
Supplement: Supplementary file 2 — Appendix S1. Regional and country specific ratings for White‐headed Vulture nest densities. [file ECE3-6-1092-s002.doc]

**Supporting Information**

**Appendix S1 - Regional and country specific ratings for White-headed Vulture nest densities.**

- Regional details:
  - West Africa. The density is based on road transect data from Thiollay (2006b, 2007) in Burkina Faso, Mali and Niger and Buij *et al.* (Buij *et al.* 2013a, Buij *et al.* 2013b, Buij and Croes 2013, 2014) in Cameroon. Road transect density is adjusted to a nest density using a correction factor (0.405) obtained from road transect to nest density ratios in Kruger National Park (South Africa), where there are high accuracy data: known number of nests and road transect density in the same area from approximately 30,000 km of road transects.
    - The West African rating for 2003/2004 is 0.00263 nests km^-2^ in West African protected areas . Other areas (i.e. unmanaged national parks, hunting reserves and other areas) given a rating of 0.0005 nests km^-2^ .
    - The 2013 West African rating is 0.00189 nests km^-2^ in protected areas and 0.0004 nests km^-2^ in other areas, and is based on a continuation of the exhibited rate of decline from 1970 to 2004 (~3.6% decline per annum).
  - East Africa –density estimation process is the same as for West African data, but the East Africa rating uses road transect data from Virani *et al.* (2011), M. Z. Virani (unpublished data) and J.-M. Thiollay, D. L. Ogada *et al*. and D. Pomeroy *et al.* (unpublished data), annualised to 2013:
    - A summarised East Africa rating – 0.0009 nests km^-2^
    - A lower rating density for outside national parks and reserves – 0.0004 nests km^-2^
  - Southern Africa -
    - Summarised National Park density based on mean nest density in Kruger National Park – 0.003 nests km^-2^
    - Low density (LD) areas, based on Kruger National Park low density area – 0.0018 nests km^-2^
- Country specific details:
  - Angola - density in all western areas reduced to zero after Thiollay . Eastern national parks and hunting areas assigned southern Africa low density rating (0.0018 nests km^-2^) as the species still occurs there , though not reported by Mills & Dean . Kangandala (09^0^53’S 16^0^44’E) excluded due to non-presence , adjacent Luando Nature Reserve (10^0^54’S 17^0^29’E) assigned minimum density (0.0001 nests km^-2^).
  - Benin – areas south of 7^0^30' excluded as out of range. National parks assigned the West African rating (0.00189 nests km^-2^). Hunting reserves assigned peripheral rating (0.0004 nests km^-2^). Confirmed as still present in Pendjari (10^0^89’N 01^0^14’E; three birds seen, March 2014, T. Helsens, *in litt*.).
  - Botswana – National parks and game reserves assigned a combined Botswana rating (0.0018 nests km^-2^) after Herremans and Herremans-Tonnoeyer (2000) and W. D. Borello (unpublished data), except arid zone parks and reserves (e.g. Gemsbok (25^0^01’S 20^0^57’E), Central Kalahari Game Reserve (22^0^36’S 23^0^49’E)) assigned minimal rating (0.0001 nests km^-2^) due to aridity. Wildlife management areas assigned a low density rating (0.0008 nests km^-2^) after W. D. Borello (unpublished data). The species has for some time been considered widespread but uncommon and occurring at low densities .
  - Burkina Faso – W National Park (11^0^53’N 02^0^07’E) assigned West Africa rating (0.00189 nests km^-2^). Faunal reserves and partial faunal reserves assigned a low density rating (0.0004 nests km^-2^). Far northern Sahel Partial Fauna Reserve ((14^0^40’N 00^0^ 30’W) not part of range map and approaching arid zone) assigned a very low rating (0.0004 nests km^-2^), unmanaged National Parks assigned very low rating (0.0004 nests km^-2^).
  - Burundi – All protected areas assigned East Africa rating (0.0009 nests km^-2^).
  - Cameroon – Areas south of 7^0^30’N excluded as out of range. All national parks assigned an adjusted West Africa rating (0.0005 nests km^-2^) after Buij and Croes and Buij *et al.* (2013a, b).
  - Central African Republic – Areas south of 7^0^30'N excluded as out of range. National Parks rated same as Kruger low density (0.0018 nests km^-2^). The species was considered rare or occasional in CAR some time ago.
  - Chad – Areas north of 17^0^N excluded as out of range and too arid. All areas assigned very low density rating (0.0001 nests km^-2^) after Wacher *et al.* . In 2003/2004 the large Ouadi Rimé-Ouadi Achim Faunal reserve (16^0^19’N 19^0^37’E) given minimal rating (0.00005 nests km^-2^), as considered out of range, but known to occur historically , although not reported by Wacher *et al.* (2013). For 2013 Ouadi Rimé-Ouadi Achim excluded and all areas assigned very low density rating (0.0001 nests km^-2^) after Wacher *et al.* (2013).
  - Côte d’Ivoire – Areas south of 7^0^30'N excluded as out of range. Faunal reserves assigned low density rating (0.0004 nests km ^-2^) , national parks assigned West African rating (0.00189 nests km^-2^) .
  - Congo, Dem. Rep. of – Most areas excluded as out of range (Congo Basin forests). National Parks assigned East African low density rating (0.0004 nests km^-2^) as edge of range and Congo forest habitat.
  - Eritrea – All areas assigned very low density (0.0004 nests km^-2^) due to aridity and edge of range.
  - Ethiopia – The bird is considered widespread, but available birding trip reports (nine reports between 2007 and 2009 plus J.-M Thiollay, unpublished data) indicate encounter rates similar to (or less than) low density areas of Kruger. National Parks assigned East Africa rating (0.0009 nests km^-2^), all other reserves and hunting areas assigned East Africa low density rating (0.0004 nests km^-2^).
  - Gambia – All protected areas included and assigned West Africa rating (0.00189 nests km^-2^), as most vulture species are reported to be still relatively abundant (C. Barlow *in litt*.) compared to adjacent countries (cf. Senegal).
  - Ghana – Areas south of 7^0^30’N excluded as out of range. National parks assigned West Africa rating (0.00189 nests km^-2^), except Digya National Park (07^0^27’N 00^0^20’W) assigned low density rating (0.0004 nests km^-2^) due to edge of range.
  - Guinea – All areas assigned West Africa rating (0.00189 nests km^-2^) apart from eastern areas - given low density rating (0.0004 nests km^-2^) as off all range maps, but included due to presence of national park and latitude.
  - Guinea-Bissau – All areas given low West Africa rating (0.0004 nests km^-2^) as the species not recorded recently .
  - Kenya – national parks and national reserves given East Africa rating (0.0009 nests km^-2^). Other areas East Africa low density (0.0004 nests km^-2^).
  - Malawi- All parks given minimum density (0.0001 nests km^-2^) except for Majete Reserve (15^0^56’S 34^0^37’E) given very low (0.0008 nests km^-2^) .
  - Mali - Areas north of 17^0^N excluded as out of range and too arid. National parks and IUCN Category I areas assigned West Africa rating (0.00189 nests km^-2^), other areas low density (0.0004 nests km^-2^).
  - Mozambique. Game reserves and hunting areas assigned the southern African low density rating (0.0018 nests km^-2^), national parks assigned the southern African rating (0.0030 nests km^-2^) except: Limpopo (23^0^18’S 31^0^57’E - adjacent to and contiguous with Kruger’s low density zone) and Niassa (12^0^01’S 37^0^23’E): included, but assigned very low density (0.0001 nests km^-2^) as the area is off range maps, but the species is known to occur there (trip reports). Older reports suggest the species ‘virtually all but disappeared’ .
  - Namibia. Game reserves and hunting areas assigned low density (0.0018 nests km^-2^), national parks assigned southern Africa rating (0.0037 nests km^-2^) except Etosha National Park (18^0^55’S 15^0^41’E) assigned low density (0.0018 nests km^-2^) due to aridity. Skeleton Coast (19^0^42’S 13^0^02’E), Namib (24^0^46’S 15^0^15’E) and southern/desert coastal areas excluded (H. Kolberg, *in litt.*, R. Simmons, *in litt.* 2011 – Namibia Red Data Book).
  - Niger – Areas north of 17^0^N excluded as out of range and too arid. The large Termit et de Tin Toumma (17^0^00’N 11^0^17’E) and Aïr Reserves (19^0^04’N 09^0^50’E) excluded due to aridity and out of range (but the species possibly occurs there). All other areas assigned very low rating (0.0004 nests km^-2^) after Petersen *et al.* and Wacher *et al*. . Very few records exist of the species away from the far south-western corner of the country (J. Brouwer, *in litt.* Niger Bird Atlas – www.WABDaB.org).
  - Nigeria – Areas south of 7^0^30'N excluded as out of range. All national parks assigned West Africa rating (0.00189 nests km^-2^). Recent (2011-2012) unpublished reports (R. Akagu, *in litt.*) suggest that many parts of Nigeria are now devoid of large vultures. Game reserves assigned a low density rating (0.0004 nests km^-2^) after old information .
  - Rwanda – All national parks assigned East Africa rating (0.0009 nests km^-2^)
  - Senegal – National parks assigned a West Africa rating (0.00189 nests km^-2^) and other areas very low rating (0.0004 nests km^-2^). This is likely an overestimate, based on very low reported densities and that almost all recent correspondence (birding trip reports and fieldworker reports from 2008 onwards) indicate that vulture numbers of all species are very low in Senegal (cf. Gambia).
  - Somalia – Western reserves assigned East Africa rating (0.0009 nests km^-2^), eastern reserves excluded as off range maps.
  - South Africa – National parks and nature reserves assigned southern Africa rating (0.0037 nests km^-2^) except: Kgalagadi Transfrontier Park (25^0^35’S 20^0^16’E) assigned minimal density (0.0008 nests km^-2^) due to aridity; Ithala Game Reserve (27^0^30’S 31^0^27’E) and Greater St Lucia/Mkuze Nature Reserve (28^0^02’S 32^0^30’E) assigned zero rating after McKean *et al.* . Other areas assigned low density (0.0018 nests km^-2^).
  - Sudan and South Sudan – Areas north of 17^0^N excluded as out of range. Unpublished reports suggest the bird occurs at low densities and is widespread, but published accounts are elusive, except Mallalieu , who reported no records of breeding. National parks assigned East Africa rating (0.0009 nests km^-2^) and other reserves the East African low density rating (0.0004 nests km^-2^).
  - Swaziland – Density in all areas reduced to zero as the species has disappeared as a breeding resident (A. Monadjem, pers. comm.).
  - Tanzania – Most game reserves given a TZ rating (0.0018 nests km^-2^), but Serengeti (02^0^29’S 34^0^47’E) and northern parks assigned 0.002 nests km^-2^ . Tanzania benefits from an established bird atlas, from which individual ratings were made for most protected areas (N. Baker, *in litt.*). Other Game reserves and Wildlife Management Areas assigned separate density ratings after Goodman .
  - Togo – Areas south of 7^0^30’N excluded as out of range. National parks assigned West Africa rating (0.00189 nests km^-2^), other areas low density (0.0004 nests km^-2^).
  - Uganda – National parks assigned East Africa rating (0.0009 nests km^-2^). Other areas low density rating (0.0004 nests km^-2^). Kibale (00^0^27’N 30^0^ 19’E), Ruwenzoris (00^0^27’N 29^0^ 57’E) and Mt Elgon (01^0^15’N 34^0^ 30’E) National parks excluded as out of range (D. Pomeroy, *in litt.*)
  - Zambia – National parks assigned southern Africa rating (0.003 nests km^-2^) as most larger parks reported to have moderate vulture populations , safari areas and wildlife management areas assigned low density (0.0018 nests km^-2^). Copperbelt, North-Western Luapula areas assigned minimal density (0.0001 nests km^-2^) based on unpublished accounts (R. McDougall, unpublished data) or off range maps.
  - Zimbabwe – National parks assigned southern Africa rating (0.003 nests km^-2^) except Hwange (19^0^13’S 26^0^30’E) assigned low density (0.0018 nests km^-2^) , safari areas and wildlife management areas assigned low density rating (0.0018 nests km^-2^).

References

Borello WD (1987) Vulture distribution in Botswana. Babbler 13, 11-25.

Bretagnolle, F. (1993) An annotated checklist of birds of north-eastern Central African Republic. Malimbus, 15, 16.

Buij, R. & Croes, B.M. (2013) Raptor habitat use in the Lake Chad Basin: insights into the effect of flood-plain transformation on Afrotropical and Palearctic raptors. Bird Conservation International, 23, 199-213.

Buij, R. & Croes, B.M. (2014) Raptors in northern Cameroon, December 2005-December 2010. Bulletin of the African Bird Club, 21, 26-63.

Buij, R., Croes, B.M. & Komdeur, J. (2013a) The role of breeding range, diet, mobility and body size in associations of raptor communities and land-use in a West African savanna. Biological Conservation, 166, 231-246.

Buij, R., Croes, B.M. & Komdeur, J. (2013b) Biogeographical and anthropogenic determinants of landscape-scale patterns in a West African raptor assemblage. Biodiversity and Conservation, 22, 1623-1646.

Carroll, R.W. (1988) Birds of the Central African Republic. Malimbus, 10, 177-200.

Goodman, P.S. (2012) Abundance of some important large birds in the Ikorongo-Grumeti Game Reserves complex: 2012. In, pp. 1-14. Unpublished Report, Grumeti Reserves, Serengeti District, Tanzania.

Goodman, P.S. (2014) Abundance of some important large birds in the Ikorongo-Grumeti Game Reserves complex: 2013. In, pp. 1-16. Unpublished Report, Grumeti Reserves, Serengeti District, Tanzania.

Green, A.A. (1983) The birds of the Bamingui-Bangoran National Park, Central African Republic. Malimbus, 5, 17-30.

Green, A.A. (1989) Avifauna of Yankari Reserve, Nigeria: new records and observations. Malimbus, 11, 61-72.

Guilherme, J.L. (2014) Birds of the Boé region, south-east Guinea-Bissau, including the first country records of Chestnut-backed Sparrow Lark Eremopterix leucotis, Lesser Striped Swallow Cecropsis abyssinica and Heuglin's Wheatear Oenanthe heuglini. Bulletin of the African Bird Club, 21, 155-168.

Herremans, M. & Herremans-Tonnoeyr, D. (2000) Land use and the conservation status of raptors in Botswana. Biological Conservation, 94, 31-41.

Hustler, K. & Howells, W.W. (1988) Breeding biology of the Whiteheaded Vulture in Hwange National Park, Zimbabwe. Ostrich, 59, 21-24.

Mallalieu, M. (2013) Bird observations around Juba, South Sudan. Bulletin of the African Bird Club, 20, 156-176.

McKean, S., Mander, M., Diedrichs, N., Ntuli, L., Mavundla, K., Williams, V. & Wakelin, J. (2013) The impact of traditional use on vultures in South Africa. Vulture News, 65, 15-36.

Mendelsohn, J. (2013) Vultures in Angola. Vulture News, 63, 71-72.

Mills, M.S.L. & Dean, W.R.J. (2013) The avifauna of the Lagoa Carumbo area, northeast Angola. Malimbus, 35, 77-92.

Mills, M.S.L., Vaz Pino, P. & Dean, W.R.J. (2008) The avifauna of Cangandala National Park, Angola. Bulletin of the African Bird Club, 15, 113-120.

Newby, J.E. (1979) The birds of the Oadi Rime - Ouadi Achim Faunal Reserve, a contribution to the study of Chadian avifauna. Malimbus, 1, 90-109.

Parker, V. (1995) The current status of some birds of prey in southern Mozambique. Journal of African Raptor Biology, 10, 2-3.

Pennycuick, C.J. (1976) Breeding of the lappet-faced and white-headed vultures (Torgos tracheliotus Forster and Trigonoceps occipitalis Burchell) on the Serengeti Plains, Tanzania. East African Wildlife Journal, 14, 67-84.

Petersen, B.S., Christensen, K.D. & Jensen, F.P. (2007) Bird population densities along two precipitation gradients in Senegal and Niger. Malimbus, 29, 101-121.

Rondeau, G. & Thiollay, J.M. (2004) West African vulture decline. Vulture News, 51, 13-31.

Roxburgh, L. & McDougall, R. (2012) Vulture poisoning incidents and the status of vultures in Zambia and Malawi. Vulture News, 62, 33-39.

Salewski, V. (2000) The birds of Comoé National Park Ivory Coast. Malimbus, 22, 55-76.

Thiollay, J.-M. (1985) The birds of Ivory Coast: Status and Distribution. Malimbus, 7, 1-59.

Thiollay, J.M. (2006a) Large bird declines with increasing human pressure in savanna woodlands (Burkina Faso). Biodiversity and Conservation, 15, 2085-2108.

Thiollay, J.M. (2006b) The decline of raptors in West Africa: long-term assessment and the role of protected areas. Ibis, 148, 240-254.

Thiollay, J.M. (2007) Raptor declines in West Africa: comparisons between protected, buffer and cultivated areas. Oryx, 41, 322-329.

Virani, M.Z., Kendall, C., Njoroge, P. & Thomsett, S. (2011) Major declines in the abundance of vultures and other scavenging raptors in and around the Masai Mara ecosystem, Kenya. Biological Conservation, 144, 746-752.

Wacher, T., Newby, J.E., Houdou, I., Harouna, A. & Rabeil, T. (2013) Vulture observations in the Sahelian zones of Chad and Niger. Bulletin of the African Bird Club, 20, 186-199.
